# Working to Increase Vaccination for Human Papillomavirus: A Survey of Wisconsin Stakeholders, 2015

**DOI:** 10.5888/pcd14.160610

**Published:** 2017-09-28

**Authors:** Sarah Mroz, Xiao Zhang, Mercedes Williams, Amy Conlon, Noelle K. LoConte

**Affiliations:** 1 University of Wisconsin Carbone Cancer Center, Madison, Wisconsin; 2 Wisconsin Comprehensive Cancer Control Program, Madison, Wisconsin; 3 University of Wisconsin Cancer Prevention and Outcomes Data Shared Resource, Madison, Wisconsin; 4 University of Wisconsin School of Medicine and Public Health, Madison, Wisconsin

## Abstract

**Introduction:**

Infection with human papillomavirus (HPV) is common and can progress to various types of cancer. HPV infection can be prevented through vaccination; however, vaccination rates among adolescents are low. The objective of this study was to assess efforts among Wisconsin stakeholders in HPV vaccination and organizational capacity for future collaborative work.

**Methods:**

We conducted a cross-sectional online survey of 277 stakeholders in HPV vaccination activities, from April 30, 2015, through June 30, 2015. Stakeholders were public health professionals, health care providers, educators, quality improvement professionals, researchers, and advocates identified as engaged in HPV vaccination work.

**Results:**

Of the 277 invited stakeholders, 117 (42%) responded to the survey. Findings showed that most current HPV vaccination activities targeted 3 groups: adolescents and parents, clinical and health professionals, and communities and health systems. The main activities directed at these groups were providing printed educational materials, professional education, and media campaigns to raise awareness. Common barriers reported were lack of understanding about the link between HPV and cancer, requests to delay vaccination, difficulty completing the 3-dose vaccine series, and reluctance to discuss sexuality.

**Conclusion:**

HPV vaccination rates are far below those of other vaccinations administered to adolescents in Wisconsin. Our study showed that various local efforts were being made to increase HPV vaccination uptake; however, many barriers exist to initiation and completion of the vaccine series. Future interventions should address barriers and employ evidence-based strategies for increasing HPV vaccination rates.

## Introduction

Human papillomavirus (HPV) infection is common, especially among young adults (1–3). Almost all cervical and anal cancers and most oropharyngeal cancers are caused by persistent infection with high-risk HPV, types 16 and 18 (4–6). HPV vaccines offer the best protection to women and men who receive all 3 doses in the series and have time to develop an immune response before becoming sexually active. Initiation of HPV vaccination is recommended for girls and boys beginning when they are aged 11 to 12 years (6). It is also recommended for girls aged 13 to 26 and for boys aged 13 to 21 who have not been vaccinated (7–9). However, since licensure of the first HPV vaccine in 2006, vaccination rates have remained low in the United States compared with rates for other adolescent vaccinations. In Wisconsin, data from the Wisconsin Immunization Registry indicate that HPV vaccination coverage is far lower than for other vaccines administered to adolescents. In 2013, an estimated 55% of girls and 29% of boys aged 13 to 17 had initiated the HPV vaccine series, and only 34% of girls and 11% of boys aged 13 to 17 had completed the series. By comparison, 92% of adolescents in this age group have received the tetanus, diphtheria, pertussis (Tdap) vaccine, and 75% had received the meningococcal conjugate vaccine (10).

The Wisconsin Comprehensive Cancer Control Program, housed at the University of Wisconsin Carbone Cancer Center, was among 18 sites funded by the National Cancer Institute to conduct an environmental scan of HPV vaccination activities to enhance collaborations for developing research and interventions to increase HPV vaccination rates in Wisconsin (11). The scan involved 4 components: 1) examination of vaccination data from the Wisconsin Immunization Registry, 2) a survey of stakeholders’ HPV-related projects, 3) assessment of current University of Wisconsin–Madison research on HPV, and 4) evaluation of a quality improvement education series for health care providers. The objective of this study was to assess local interventions to increase HPV vaccination and organizational capacity for future collaborative work in Wisconsin.

## Methods

### Study population

We conducted a cross-sectional online survey of 277 Wisconsin stakeholders in HPV vaccination activities from April 30, 2015, through June 30, 2015 (Appendix). Stakeholders were public health professionals, health care providers, educators, quality improvement professionals, researchers, and advocates from the state who attended or were invited to the Wisconsin HPV Vaccine Summit in June of 2014. The summit was a conference designed to mobilize vaccine advocates by providing data on state-specific HPV disease epidemiology and immunization rates, updates on the diseases caused by HPV, vaccine safety information, and discussions on how to address vaccination hesitancy and strengthen vaccination recommendations. We also surveyed members of the 15 local or regional immunization coalitions in Wisconsin. Stakeholders received an email inviting them to participate in the online survey. Four follow-up email reminders were sent to incomplete survey respondents and nonrespondents at intervals of every 2 weeks to encourage participation. 

### Measures

Study participants reported on their role in their organization and answered other organization-related questions, including type of organization, whether the organization belonged to a local or regional immunization coalition, whether the organization accessed the Wisconsin Immunization Registry, and whether the organization used the Registry for Effectively Communicating Immunization Needs and an electronic health record to track immunizations. Respondents were also asked about their organization’s interest in conducting new activities and their capacity to take on new activities to promote HPV vaccination.

Respondents were asked whether their organizations conducted any activities in Wisconsin in 2013, 2014, or 2015 related to HPV vaccination. The activities were classified into 4 categories:1) adolescents and their parents, 2) clinical and health care professionals, 3) communities and health systems, and 4) advocacy and public policy. If respondents reported activities in any year in any of these categories, they were asked to provide information about how often they encountered certain barriers (never, rarely, sometimes, often, or always). The selections of sometimes, often, and always were defined as barriers. In addition, respondents provided information on their funding sources and collaborating organizations for each category of activities.

### Statistical analysis

We calculated simple descriptive statistics to examine the prevalence of HPV vaccination-related activities that stakeholders conducted from 2013 through 2015 and the barriers they encountered. We then tested for trends in different activities over time. All analyses were performed with Stata/MP version 13.1 (StataCorp LP). Significance was set at *P* less than .05.

## Results

Of the 277 stakeholders we invited to participate, 117 replied, a response rate of 42.2%. Of the 117 stakeholders who participated in the survey, 37 (31.6%) classified themselves as public health professionals, 21 (17.9%) as health care providers, and 9 (7.7%) as educators (Table). Thirty (25.6%) of the respondents worked for local public health departments, 8 (6.8%) worked for state health departments, and 22 (18.8%) for health care providers/health systems (categories were not mutually exclusive). Fifty-six (47.9%) indicated that they or their organization belonged to a local or regional immunization coalition, 71 (60.7%) reported that their organizations currently accessed the Wisconsin Immunization Registry, 6 (5.1%) used the Regional Early Childhood Immunization Network, and 32 (27.4%) used an electronic health record to track immunizations. Over half of respondents 67 (57.3%) expressed a medium to high level of interest in conducting new activities to promote HPV vaccination. However, only 46 (39.3%) rated their capacity to take on new activities as adequate (medium or high capacity).

**Table:** Characteristics of HPV Vaccination Stakeholders and Their Organizations in Wisconsin (N = 117)

Characteristic	No. (%)
**Position or role in organization^a^**	
Public health professional	37 (31.6)
Health care provider	21 (17.9)
Educator	9 (7.7)
Advocate	6 (5.1)
Member of a community-based organization	7 (6.0)
Researcher/academic staff	6 (5.1)
Other or unknown	51 (43.6)
**Type of organization^a^**	
Local public health department	30 (25.6)
State public health department	8 (6.8)
Health care provider/health system	22 (18.8)
College or university	11 (9.4)
Other or unknown	50 (42.7)
**Belong to a local or regional immunization coalition**	56 (47.9)
**Currently access the Wisconsin Immunization Registry**	71 (60.7)
**Currently use the Regional Early Childhood Immunization Network to track immunizations**	6 (5.1)
**Currently use an electronic health record to track immunization**	32 (27.4)
**Interest level in conducting new activities to promote HPV vaccination**	
High	40 (34.2)
Medium	27 (23.1)
Low	8 (6.8)
No	3 (2.6)
Don’t know	39 (33.3)
**Capacity to take on new activities to promote HPV vaccination**	
High	7 (6.0)
Medium	39 (33.3)
Low	24 (20.5)
No	3 (2.6)
Don’t know	44 (37.6)
**Have at least one staff member serving as a lead for a project to increase HPV vaccination rates**	55 (47.0)

Abbreviation: HPV, human papillomavirus.

a Categories are not mutually exclusive.

**Figures 1a–d:**
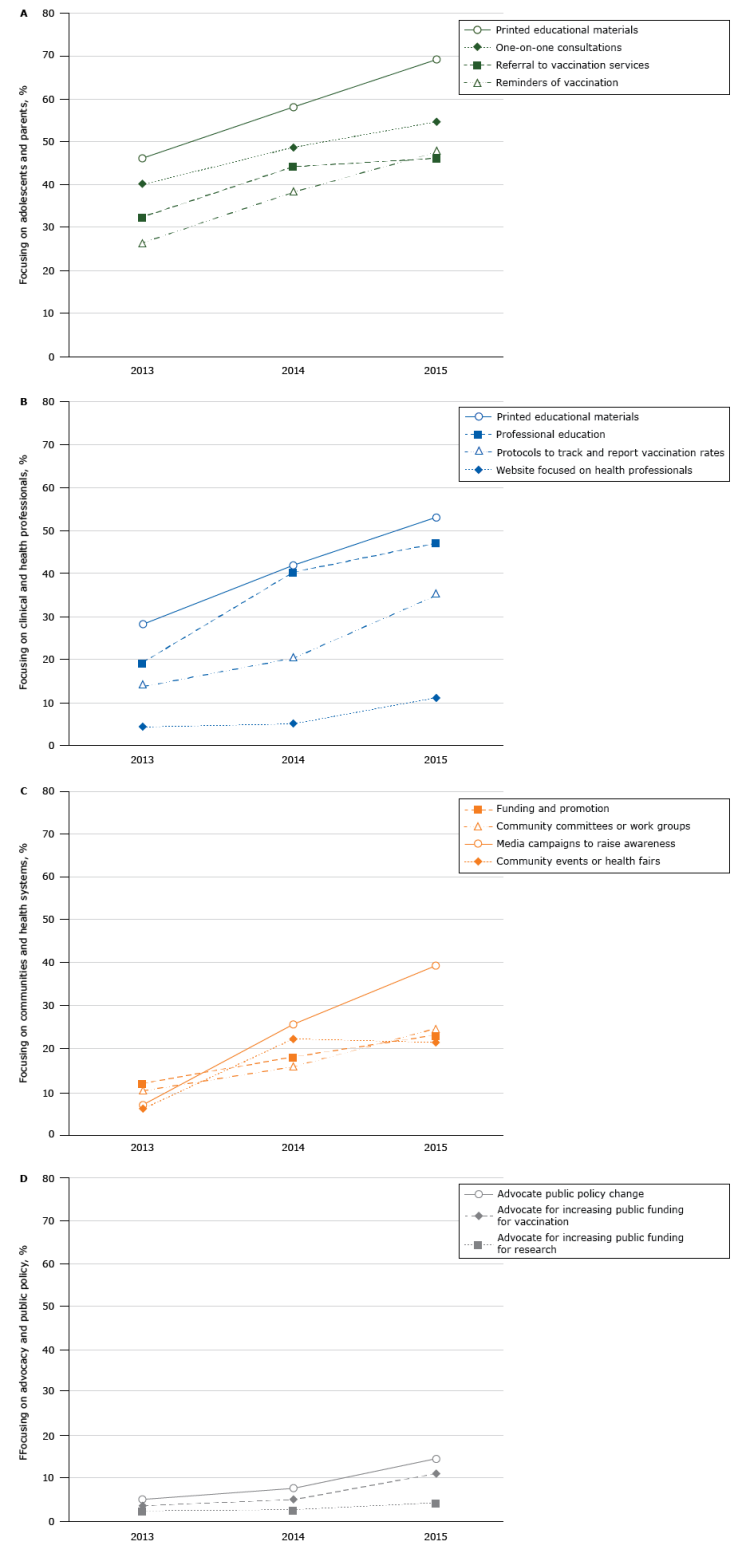
Percentage of Wisconsin stakeholder organizations (N = 117) reporting activities to increase human papillomavirus vaccination in Wisconsin, 2013–2015. Figure 1a shows stakeholder organizations with activities focused on adolescents and parents, 1b shows stakeholder organizations with activities focused on clinical and health professionals, 1c shows stakeholder organizations with activities focused on communities and health systems, and 1d shows stakeholder organizations with activities focused on advocacy and public policy. The rates of advocating for public policy change and advocating for increased public funding increased over time (*P* < .05). **Table:** 

Stakeholders reported on activities that their organizations conducted in recent years to increase HPV vaccination. Ninety-four (80.3%) reported that their organizations were involved in activities focusing on adolescents (girls or boys aged 11–18 y) and their parents. In 2015, the most common activities involving adolescents and their parents were providing printed educational materials (69.2%), one-on-one consultations to adolescents and parents on HPV vaccination (54.7%), reminders to adolescents and parents of when adolescents were due for HPV vaccination (47.9%), and referrals to HPV vaccination services (46.2%). The pattern was similar for 2013 and 2014, and the prevalence of these 4 types of activities increased significantly over time (*P* < .05) (Figure 1a).

Stakeholder Target Audience	2013	2014	2015
	Activity Rate, %		
**1a. Adolescents and parents **			
Printed educational materials	46.2	58.1	69.2
One-on-one consultations	41.0	48.7	54.7
Reminders of vaccination	26.5	38.5	47.9
Referrals to vaccination services	32.5	44.4	46.2
**1b. Clinical and health professionals**			
Printed educational materials	28.2	41.9	53.0
Professional education	19.7	40.2	47.0
Protocols to track and report vaccination rates	13.7	20.5	35.0
Website focused on health professionals	4.3	5.1	11.1
**1c. Communities and health systems**			
Community events or health fairs	6.0	22.2	21.4
Media campaigns to raise awareness	6.8	25.6	39.3
Community committees or work groups	10.3	16.2	24.8
Funding and promotion	12.0	17.9	23.1
**1d. Advocacy and public policy**			
Advocate public policy change	5.1	7.7	14.5
Advocate for increasing public funding for vaccination	3.4	5.1	11.1
Advocate for increasing public funding for research	2.6	2.6	4.3

**Figures 2a–d:**
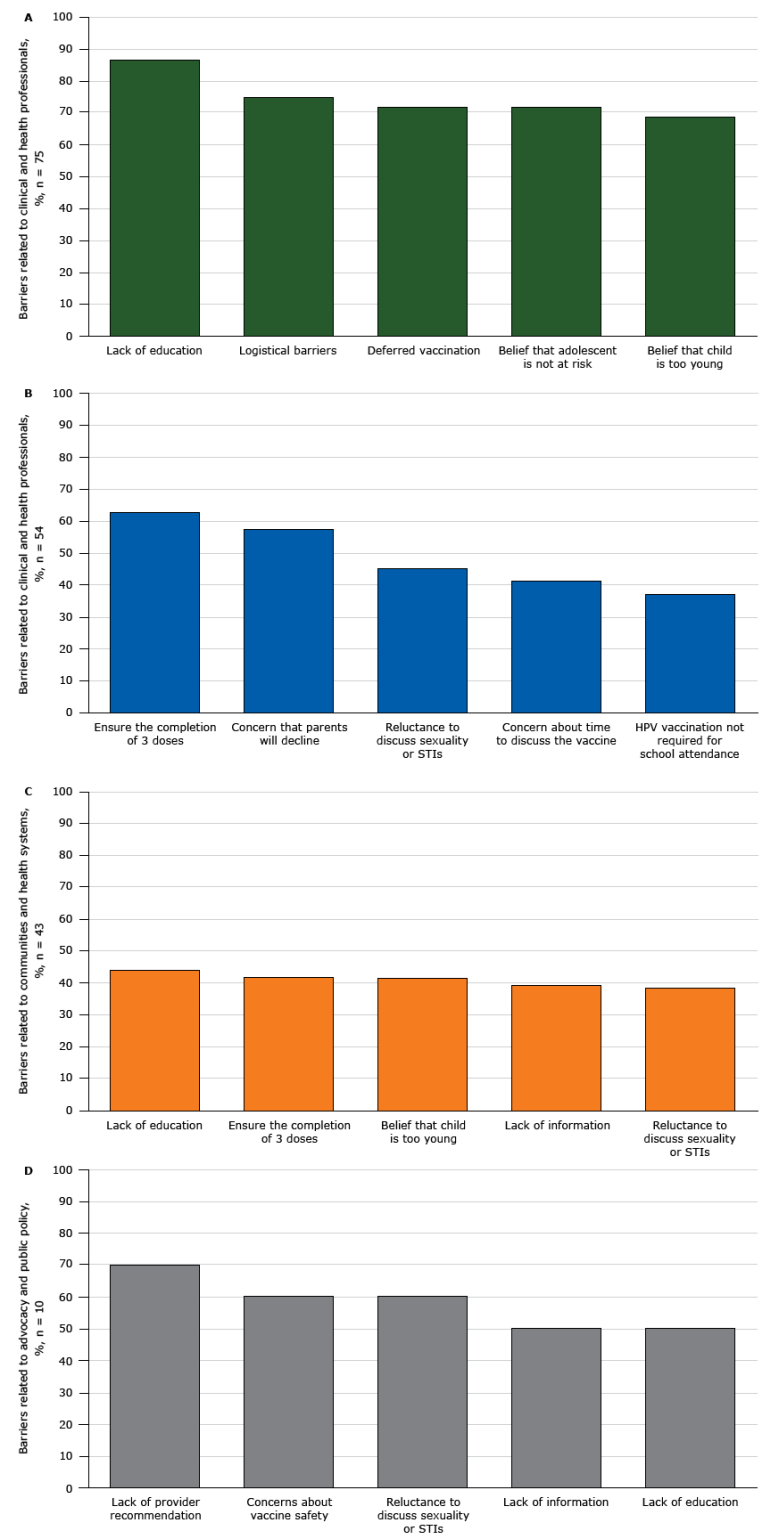
Percentage of Wisconsin stakeholder organizations (N = 117) reporting barriers to human papillomavirus (HPV) vaccination, from 2013 through 2015. Figure 2a shows percentage of stakeholder organizations reporting barriers related to adolescents and parents (n = 75), 2b shows the percentage of stakeholder organizations reporting barriers related to clinical and health professionals (n = 54), 2c shows the percentage of stakeholder organizations reporting barriers related to communities and health systems (n = 43), and 2d shows the percentage of stakeholder organizations reporting barriers related to advocacy and public policy (n = 10). Abbreviation: STI, sexually transmitted infection. **Table:** 

Overall, 75 respondents reported barriers to activities focusing on adolescents and parents. The most common barriers were lack of education or understanding about HPV infection, including its link to cancer (86.6%); logistical or other barriers to returning for the full series of 3 shots (74.7%); requests that HPV vaccination be deferred (71.7%); belief that the adolescent is not at risk for HPV infection (71.6%); and the parent’s belief that child is too young for the HPV vaccine (68.5%, Figure 2).

Category	%
**2a. Adolescents and parents, n = 75**	
Lack of education	86.6
Logistical barriers	74.7
Deferred vaccination	71.7
Belief that adolescent is not at risk	71.6
Belief that child is too young	68.5
**2b. Clinical and health professionals, n = 54**	
Ensure the completion of 3 doses	63.0
Concern that parents will decline	57.7
Reluctance to discuss sexuality or STIs	45.3
Concern about time to discuss the vaccine	41.5
HPV vaccination not required for school attendance	37.1
**2c. Communities and health systems, n = 43**	
Lack of education	44.1
Ensure the completion of 3 doses	41.9
Belief that child is too young	41.8
Lack of information	39.5
Reluctance to discuss sexuality or STIs	38.7
**2d. Advocacy and public policy, n = 10**	
Lack of provider recommendation	70.0
Concerns about vaccine safety	60.0
Reluctance to discuss sexuality or STIs	60.0
Lack of information	50.0
Lack of education	50.0

Seventy-six of the 117 respondents (65.0%) indicated that their organizations were conducting activities focusing on clinical and health professionals to increase HPV vaccination. The most commonly reported activities in 2015 were providing printed educational materials (53.0% of stakeholders), professional education on HPV vaccination (47.0%), protocols to track and report on HPV vaccination rates (35.0%), and websites focused on health professionals (11.1%) (Figure 1b). The increase in all activity rates from 2013 to 2015 was significant (*P* < .05). Barriers most often encountered by these organizations (N = 54) were difficulty ensuring that patients completed the 3-dose HPV vaccination series (63.0%), concern that parents would decline HPV vaccination despite appropriate counseling (57.7%), reluctance to discuss sexuality or sexually transmitted infections (45.3%), concern about the time it takes to discuss HPV vaccination with patients or parents (41.5%), and HPV vaccination not being required by schools (37.1%) (Figure 2b).

Sixty-one stakeholders (52.1%) identified their organization’s activities as projects focusing on communities and health systems. The most commonly reported activity in this category in 2015 was media campaigns to raise awareness about the need for HPV vaccination of adolescents (39.3%,), followed by community committee or work groups that focus on HPV vaccination (24.8%), support for community groups that fund and promote the vaccine (23.1%), and community events or health fairs to promote HPV vaccination (21.4%) (Figure 1c). The increase from 2013 through 2015 in these activities was significant (*P* < .05). Barriers experienced in implementing community- and health-system–focused activities (N = 43) were lack of education or understanding about HPV infection, including its link to cancer (44.1%); difficulty ensuring that patients will complete the 3-dose HPV vaccination series (41.9%); belief that younger adolescents are too young for the HPV vaccinations (41.8%); and reluctance to discuss sexuality or sexually transmitted infections (38.7%) (Figure 2c).

Lastly, 18 respondents (15.4%) indicated that they were involved in activities focused on advocacy and public policy with the most commonly reported activity in 2015 being efforts to increase HPV vaccination rates through advocating public policy change (17 respondents), followed by advocating for increasing public funding for HPV vaccination (13 respondents) and advocating for increased public funding for HPV research (5 respondents) (categories were not mutually exclusive). The rates of the former 2 activities increased over time (*P* < .05) (Figure 1d). Barriers to these activities that respondents (N = 10) reported were described as lack of provider recommendations for HPV vaccination (7 respondents), concerns about vaccine safety (6 respondents), and reluctance to discuss sexuality or sexually transmitted infections (6 respondents) (Figure 2d)

Most organizations reported working with partners on activities promoting HPV vaccination: activities that focused on adolescents and parents (50 of 94 respondents), activities that focused on clinical and health professionals (47 of 76 respondents), activities that focused on communities and health systems (37 of 61 respondents), and activities that focused on advocacy and public policy (14 of 18 respondents). Commonly reported collaborators were the Centers for Disease Control and Prevention (CDC), the Wisconsin Immunization Program, local health departments, local clinics and hospitals, schools, local immunization coalitions, the Wisconsin Comprehensive Cancer Control Program, and the Wisconsin chapter of the American Academy of Pediatrics. Primary sources of funding for HPV vaccination activities were CDC, the Wisconsin chapter of the American Academy of Pediatrics, the Wisconsin Department of Health Services, the University of Wisconsin Partnership Program, the Wisconsin Immunization Program, immunization coalitions, the Wisconsin Comprehensive Cancer Control Program, and tax levy funds.

## Discussion

Persistent HPV infection can lead to cancer of the cervix, vulva, vagina, penis, anus, and oropharynx (12). The HPV vaccine is safe and effective and has been recommended since 2006 (13). However, HPV vaccination coverage has remained far below that of other vaccinations administered to adolescents, in Wisconsin and throughout the United States. To assess efforts to increase HPV vaccination in Wisconsin and barriers encountered, we conducted a survey of stakeholders involved in the effort. Findings from the survey illustrate the HPV vaccination-related projects being conducted in Wisconsin and provide a roadmap for future collaboration and activities to increase HPV vaccination.  Our survey of stakeholder activities found that ongoing work is focused on 4 main groups: adolescents and parents, clinical and health professionals, communities and health systems, and stakeholders involved with advocacy and public policy. HPV vaccination promotion activities increased across all these groups from 2013 through 2015. Many survey respondents reported conducting activities that were focused on adolescents and parents, the most common of which were the provision of educational materials, one-on-one consultations and referrals, and reminders for vaccination. The main activities used to influence clinical and health professionals were provision of educational materials, professional education, and protocols to track and report vaccination rates. These activities are in line with research, which indicates that educational campaigns directed at health care professionals and parents are key facilitators for promoting HPV vaccination (14). Examples of educational efforts to raise awareness about HPV and HPV vaccination are media campaigns, printed materials, online information, webinars, conferences, and in-person training sessions. Of the activities designed to affect communities and health systems, media campaigns to raise awareness were used most often, followed by health fairs and community group collaborations. We identified fewer activities in the area of advocacy and public policy, but among the activities reported were advocating for public policy change and advocating for increased public funding for vaccination. HPV vaccination promotion activities in Wisconsin are similar to efforts conducted at the health care practice or community level in other parts of the United States. The effectiveness of interventions to increase HPV vaccination has varied, and further research is needed (15–17). 

As in other research, Wisconsin stakeholders reported several main barriers to vaccination across groups: lack of understanding about HPV and its link to cancer, reluctance to discuss sexuality, parental resistance to the vaccination, belief that the adolescent is too young or is not at risk, difficulty with completion of the 3-dose vaccine series, safety concerns, and concern about promoting risky sexual behavior (3,18,19). Health care providers have been identified as uniquely positioned to address barriers perceived by parents. They can initiate conversations to address concerns and misunderstandings and recommend the vaccine (19). Studies have shown that parents and patients want additional information about HPV and the HPV vaccine and that they trust the information provided by health care providers (20). Providers need additional training on how to give a strong, clear recommendation and communicate the urgency and importance of HPV vaccination (14). Other potential strategies for providers and health care systems to increase HPV vaccination rates are reducing missed clinical opportunities and administering the HPV vaccine at the same time as other adolescent vaccines (18). To reduce the number of HPV-related cancer deaths in Wisconsin and reach the Healthy People 2020 goal of 80% HPV vaccination coverage, continued efforts are needed to cultivate provider champions for the vaccine, educate the public about its safety and efficacy, increase access to vaccination, and reduce missed clinical opportunities to vaccinate (21). The consistency of barriers cited across target groups related to the lack of understanding about HPV, the HPV vaccine, and HPV related cancers indicates that future efforts should focus on enhanced education and communication strategies for providers, parents, adolescents, and communities.   The Advisory Committee on Immunization Practices recommends that young adolescents (aged 9–14 y) move from a 3-dose to a 2-dose HPV vaccine schedule (22,23). This change presents new opportunities for increasing adherence. The change, also recommended by CDC, was based on a review of clinical trials showing that 2 doses of the vaccine among young adolescents produced an immune response similar to, or higher than, the response in young adults (aged 16–26 y) who received 3 doses (22). Because survey respondents cited logistical challenges to returning for the series of 3 shots as a barrier, the move to a 2-dose schedule — which still provides safe, long lasting, effective protection — may increase acceptance and improve vaccination rates among young adolescents (23). More than half of survey respondents expressed interest in conducting new activities to promote HPV vaccination. However, because less than 40% said they had the organizational capacity to do so, policy efforts should focus on increasing funding for HPV vaccination education and promotion. HPV vaccination rates in Wisconsin have increased in recent years. In 2015, the overall vaccination rate for adolescents aged 13 to 18 years was 44%, and in 2016 the rate was 49% (https://www.dhs.wisconsin.gov/immunization/data.htm).

A strength of our study is that it is the first statewide, comprehensive review of HPV vaccination activities in Wisconsin. A limitation is that our list of Wisconsin HPV vaccination stakeholders may be incomplete, and we do not have information about HPV vaccination-related activities conducted by stakeholders who were not identified. However, the participant list was reviewed by representatives of the Wisconsin Immunization Program, the Wisconsin chapter of the American Academy of Pediatrics, and the University of Wisconsin Immunization Taskforce to identify missing stakeholders engaged in current or recent HPV vaccination work who were then added to the group. Another limitation is that the survey response rate was moderate (42.2%), but it was within the range (40%–60%) recommended for surveys to inform key policies and resource allocation (24). A comparison between survey respondents and nonrespondents indicated that the geographic distribution was similar between the 2 groups, suggesting that the survey findings were representative of the state.  The Wisconsin HPV stakeholder survey found that activities promoting HPV vaccination are wide-ranging and have increased over time across the state. Future interventions should address identified barriers and employ evidence-based strategies for increasing HPV vaccination rates.

## Appendix Stakeholder Survey

This appendix is available for download and a Microsoft Word document.
