# Supplementary material for: Working to Increase Vaccination for Human Papillomavirus: A Survey of Wisconsin Stakeholders, 2015
Source: Prev Chronic Dis. 2017 Sep 28;14:E85. doi: 10.5888/pcd14.160610 (PMC5783328; doi:10.5888/pcd14.160610)
Supplement: Supplementary file 1 [file 16_0610Appendix.docx]

# Appendix. Stakeholder Survey

The Wisconsin Comprehensive Cancer Control Program (WI CCC Program) and the University of Wisconsin Carbone Cancer Center are working to identify organizations conducting activities focused on increasing HPV vaccination rates in Wisconsin. HPV vaccination is underutilized despite the overwhelming evidence for its safety and effectiveness. Increasing HPV vaccination is a national priority.

You or your organization has been identified as potentially engaged in current or recent HPV vaccination work. We would like to ask you some questions about HPV vaccine-related activities including those focused on patients, families, providers, communities or health systems, as well as advocacy and policy.

We would also like to know a little about your organization and your interest in and capacity to work on HPV vaccine utilization in the future. There is continued interest in developing further HPV related collaborations, and providing additional support to organizations. Completing this survey will help ensure that all potential partners are identified.

A. [FOR THOSE WHO ATTENDED THE SUMMIT AND COMPLETED THE “3 THINGS” PART OF THE EVALUATION] According to our records, you attended the Wisconsin HPV Summit in June 2014 at the Madison Concourse Hotel. When you completed the Summit evaluation, we asked you to list three things that you or your organization could do to promote the HPV vaccine. We would like to know which of the things you were able to complete.

**1. You indicated that you would “[Activity 1 fill in from response].” Were you able to complete this activity?**

No. I was not able to do any work on this activity.

No. I worked on this activity, but did not complete it.

Yes, I completed this activity.

**2. You indicated that you would “[Activity 2 fill in from response].” Were you able to complete this activity?**

No. I was not able to do any work on this activity.

No. I worked on this activity, but did not complete it.

Yes, I completed this activity.

**3. You indicated that you would “[Activity 2 fill in from response].” Were you able to complete this activity?**

No. I was not able to do any work on this activity.

No. I worked on this activity, but did not complete it.

Yes, I completed this activity.

**B.** [FOR EVERYONE] **We would like to ask you some questions about whether your organization has conducted any activities focusing on adolescents (girls or boys ages 11-18) and their parents that may increase HPV vaccination.**

Below is a list of activities focusing on adolescents and their parents that may increase HPV vaccination. Please indicate which activities, if any, your organization used (or plans to use) in 2013, 2014 or 2015.

**4. Activities that focus on Adolescents (i.e. ages 11-18) and Their Parents**

|  | No work in this area | 2013 | 2014 | 2015 |
| --- | --- | --- | --- | --- |
| a. Provide printed educational materials (brochures, etc.). | ❑ | ❑ | ❑ | ❑ |
| b. Provide and maintain an HPV immunization website focused on adolescents and parents. | ❑ | ❑ | ❑ | ❑ |
| c. Provide referrals to HPV vaccination services. | ❑ | ❑ | ❑ | ❑ |
| d. Provide one-on-one consultations to adolescents and parents on HPV vaccination. | ❑ | ❑ | ❑ | ❑ |
| e. Provide free or reduced-cost HPV vaccination. | ❑ | ❑ | ❑ | ❑ |
| f. Provide financial incentives (e.g. gift cards) for completing HPV vaccination. | ❑ | ❑ | ❑ | ❑ |
| g. Provide in-home HPV vaccination. | ❑ | ❑ | ❑ | ❑ |
| h. Provide alternate site HPV vaccination (school clinics, WIC offices, pharmacies, etc.). | ❑ | ❑ | ❑ | ❑ |
| i. Provide reminders to adolescents and parents of when adolescents are due for HPV vaccination. | ❑ | ❑ | ❑ | ❑ |
| j. Other ___________________ __________________________ | ❑ | ❑ | ❑ | ❑ |

**5. You indicated that your organization provides educational materials about HPV vaccine to adolescents and parents. What is the source of these materials?** (This question is only displayed based on the response in to 4a. above.)

🔾 CDC HPV educational materials in their original form.

🔾 CDC HPV educational materials modified to meet your organization's needs.

🔾 Educational materials developed by your organization.

🔾 Other ____________________

[DISPLAY IF ANY ACTIVITIES MARKED IN Q4 ABOVE:]

**6. We are interested in how you have funded the HPV vaccination activities focused on adolescents and their parents that you indicated above. Below, please describe any federal or state agency, non-profit organization or other sources of funding for these activities. Please note which activities and year(s) (2013, 2014, or 2015) the funding is for.**

|  |
| --- |

[DISPLAY IF ANY ACTIVITIES MARKED IN Q4 ABOVE:]

**7**

🔾 No, we did NOT have partners or collaborators; my organization did this work on our own

🔾 Yes, we did have partners or collaborators. Please specify the names of these partners or collaborators and the activities on which they worked with you:

|  |
| --- |

[DISPLAY IF ANY ACTIVITIES MARKED IN Q4 ABOVE:]

**8. When you implemented the activities focused on adolescents and their parents that you indicated above, how often did you or your organization encounter any of the following barriers?**

|  | **Never**  0% | **Rarely**  1-25% | **Some-times**  26-50% | **Often**  51%-75% | **Always**  >75% |
| --- | --- | --- | --- | --- | --- |
| a. Concerns about vaccine safety or side effects | O | O | O | O | O |
| b. Concerns about vaccine efficacy | O | O | O | O | O |
| c. Lack of knowledge among families that vaccine is a series of three shots | O | O | O | O | O |
| d. Logistical or other barriers to returning for series of three shots | O | O | O | O | O |
| e. Reluctance to discuss sexuality or sexually transmitted infections | O | O | O | O | O |
| f. Concern that adolescent will assume that a parent who agrees to HPV vaccination condones premarital sex | O | O | O | O | O |
| g. Concern that vaccinated child will practice riskier sexual behaviors | O | O | O | O | O |
| h. Lack of education/understanding about HPV infection including its link to cancer | O | O | O | O | O |
| i. Requests that HPV vaccination be deferred | O | O | O | O | O |
| j. Belief that adolescent is not at risk for HPV infection | O | O | O | O | O |
| k. Parent won’t consent to vaccination | O | O | O | O | O |
| l. Parent believes child is too young for the HPV vaccination | O | O | O | O | O |
| m. Concern about negative media reports related to the HPV vaccine | O | O | O | O | O |
| n. Lack of provider recommendations for HPV vaccination | O | O | O | O | O |
| o. Cost of HPV vaccine for family | O | O | O | O | O |
| p. Belief that HPV vaccine is recommended only for girls | O | O | O | O | O |
| q. Belief that girls or women should be the ones to take preventative steps against cervical cancer | O | O | O | O | O |

**Did you encounter any other barriers besides those just mentioned?**

|  |
| --- |

**C. Now we would like to ask you some questions about your activities focusing on clinical and health professionals that may increase HPV vaccination.**

**9. Below is a list of activities focusing on clinical and health professionals that may increase HPV vaccination. Please indicate which activities your organization used (or plan to use) in 2013, 2014 or 2015.**

|  | No work in this area | 2013 | 2014 | 2015 |
| --- | --- | --- | --- | --- |
| a. Convened or helped coordinate community events or health fairs to promote or administer HPV vaccination. | ❑ | ❑ | ❑ | ❑ |
| b. Support media campaigns to raise awareness of the need for HPV vaccination of adolescents. | ❑ | ❑ | ❑ | ❑ |
| c. Staff community committees, work groups, or roundtables that focus on increasing HPV vaccination. | ❑ | ❑ | ❑ | ❑ |
| d. Support community groups/collaborations that fund the promotion of HPV vaccination. | ❑ | ❑ | ❑ | ❑ |
| e. Convened or helped coordinate community events or health fairs to promote or administer HPV vaccination. | ❑ | ❑ | ❑ | ❑ |
| f. Other ___________________ __________________________ | ❑ | ❑ | ❑ | ❑ |

[DISPLAY IF ANY ACTIVITIES MARKED IN Q9 ABOVE:]

**10. We are interested in how you have funded the HPV vaccination activities focused on clinical and health professionals that you indicated above. Below, please describe any federal or state agency, non-profit organization or other sources of funding for these activities. Please note which activities and year(s) (2013, 2014, or 2015) the funding is for.**

|  |
| --- |

[DISPLAY IF ANY ACTIVITIES MARKED IN Q9 ABOVE:]

**11. Did you have partners or collaborating organizations for the HPV vaccination activities focused on clinical and health professionals that you indicated above? These may include any federal or state agencies, non-profit organizations, local health departments, health systems or clinics, advocacy organizations, state or local cancer or immunization coalitions, pharmacies, pharmaceutical companies or other organizations.**

🔾 No, we did NOT have partners or collaborators; my organization did this work on our own

🔾 Yes, we did have partners or collaborators. Please specify the names of these partners or collaborators and the activities on which they worked with you:

|  |
| --- |

[DISPLAY IF ANY ACTIVITIES MARKED IN Q9 ABOVE:]

**12. When you implemented the activities focused on clinical and health professionals that you indicated above, how often did you or your organization encounter any of the following barriers?**

|  | **Never**  0% | **Rarely**  1-25% | **Some-times**  26-50% | **Often**  51%-75% | **Always**  >75% |
| --- | --- | --- | --- | --- | --- |
| a. Concerns about vaccine safety | O | O | O | O | O |
| b. Concerns about vaccine efficacy | O | O | O | O | O |
| c. Reluctance to discuss sexuality or sexually transmitted infections | O | O | O | O | O |
| d. Concerns that vaccinated adolescents will practice riskier sexual behaviors | O | O | O | O | O |
| e. Beliefs that younger adolescents are too young for the vaccine | O | O | O | O | O |
| f. Concerns about administering a new vaccine with a limited track record of safety | O | O | O | O | O |
| g. Concerns about adding another vaccine to the vaccine schedule | O | O | O | O | O |
| h. Lack of information about the HPV vaccine or HPV infection, including its link to cancer | O | O | O | O | O |
| i. Concerns about the up-front cost of purchasing private stock HPV vaccine | O | O | O | O | O |
| j. Concerns about the lack of adequate reimbursement for HPV vaccination | O | O | O | O | O |
| k. Concerns about the failure of some insurance companies to cover the cost of vaccination | O | O | O | O | O |
| l. Concern about the time it takes to discuss HPV vaccination with patients and/or parents | O | O | O | O | O |
| m. Concern that parents will decline HPV vaccination despite appropriate counseling | O | O | O | O | O |
| n. Difficulty ensuring that patients will complete the 3-dose HPV vaccination series | O | O | O | O | O |
| o. Barriers due to HPV vaccination not being required for school attendance | O | O | O | O | O |
| p. Belief that HPV vaccine is recommended only for girls | O | O | O | O | O |
| q. Belief that girls or women should be the ones to take preventative steps against cervical cancer | O | O | O | O | O |

**Did you encounter any other barriers besides those just mentioned?**

|  |
| --- |

**D. Now we would like to ask you some questions about your activities focusing on communities and health systems that may increase HPV vaccination.**

**13. Below is a list of activities focusing on communities and health systems that may increase HPV vaccination. Please indicate which activities your organization used (or plan to use) in 2013, 2014 or 2015.**

|  | No work in this area | 2013 | 2014 | 2015 |
| --- | --- | --- | --- | --- |
| a. Convened or helped coordinate community events or health fairs to promote or administer HPV vaccination. | ❑ | ❑ | ❑ | ❑ |
| b. Support media campaigns to raise awareness of the need for HPV vaccination of adolescents. | ❑ | ❑ | ❑ | ❑ |
| c. Staff community committees, work groups, or roundtables that focus on increasing HPV vaccination. | ❑ | ❑ | ❑ | ❑ |
| d. Support community groups/collaborations that fund the promotion of HPV vaccination. | ❑ | ❑ | ❑ | ❑ |
| e. Other __________________ __________________________ | ❑ | ❑ | ❑ | ❑ |

[DISPLAY IF ANY ACTIVITIES MARKED IN Q13 ABOVE:]

**14. We are interested in how you have funded the HPV vaccination activities focused on communities and health systems that you indicated above. Below, please describe any federal or state agency, non-profit organization or other sources of funding for these activities. Please note which activities and year(s) (2013, 2014, or 2015) the funding is for.**

|  |
| --- |

[DISPLAY IF ANY ACTIVITIES MARKED IN Q13 ABOVE:]

**15. Did you have partners or collaborating organizations for the HPV vaccination activities focused on communities and health systems that you indicated above? These may include any federal or state agencies, non-profit organizations, local health departments, health systems or clinics, advocacy organizations, state or local cancer or immunization coalitions, pharmacies, pharmaceutical companies or other organizations.**

🔾 No, we did NOT have partners or collaborators; my organization did this work on our own

🔾 Yes, we did have partners or collaborators. Please specify the names of these partners or collaborators and the activities on which they worked with you:

|  |
| --- |

[DISPLAY IF ANY ACTIVITIES MARKED IN Q13 ABOVE:]

**16. When you implemented the activities focused on communities and health systems that you indicated above, how often did you or your organization encounter any of the following barriers?**

|  | **Never**  0% | **Rarely**  1-25% | **Some-times**  26-50% | **Often**  51%-75% | **Always**  >75% |
| --- | --- | --- | --- | --- | --- |
| a. Concerns about vaccine safety | O | O | O | O | O |
| b. Concerns about vaccine efficacy | O | O | O | O | O |
| c. Reluctance to discuss sexuality or sexually transmitted infections | O | O | O | O | O |
| d. Concern that adolescent will assume that a parent who agrees to HPV vaccination condones premarital sex | O | O | O | O | O |
| e. Concern that vaccinated adolescents will practice riskier sexual behaviors | O | O | O | O | O |
| f. Lack of education/understanding about HPV infection including its link to cancer | O | O | O | O | O |
| g. Lack of information about the HPV vaccine | O | O | O | O | O |
| h. Belief that adolescents are not at risk for HPV infection | O | O | O | O | O |
| i. Belief that younger adolescents are too young for the HPV vaccination | O | O | O | O | O |
| j. Lack of knowledge among families that vaccine is a series of three shots | O | O | O | O | O |
| k. Concerns about logistical or other barriers to returning for series of three shots | O | O | O | O | O |
| l. Lack of provider recommendations for HPV vaccination | O | O | O | O | O |
| m. Belief that HPV vaccine is recommended only for girls | O | O | O | O | O |
| n. Belief that girls or women should be the ones to take preventative steps against cervical cancer | O | O | O | O | O |
| o. Concerns about adding another vaccine to the vaccine schedule | O | O | O | O | O |
| p. Cost of HPV vaccine for family | O | O | O | O | O |
| q. Concerns about the up-front cost of purchasing private stock HPV vaccine | O | O | O | O | O |
| r. Concerns about the lack of adequate reimbursement for HPV vaccination | O | O | O | O | O |
| s. Difficulty ensuring that patients will complete the 3-dose HPV vaccination series | O | O | O | O | O |
| t. Barriers due to HPV vaccination not being required for school attendance | O | O | O | O | O |

**Did you encounter any other barriers besides those just mentioned?**

|  |
| --- |

**E. Now we would like to ask you some questions about your activities focusing on advocacy and public policy that may increase HPV vaccination.**

**17. Below is a list of activities focusing on advocacy and public policy that may increase HPV vaccination. Please indicate which activities your organization used (or plan to use) in 2013, 2014 or 2015. Provide a brief description of each activity you have used or plan to use**.

|  | No work in this area | 2013 | 2014 | 2015 |
| --- | --- | --- | --- | --- |
| a. Support efforts to increase HPV vaccination rates through advocating public policy change. | ❑ | ❑ | ❑ | ❑ |
| b. Advocate for increasing public funding for HPV vaccination. | ❑ | ❑ | ❑ | ❑ |
| c. Advocate for increasing public funding for HPV research. | ❑ | ❑ | ❑ | ❑ |
| d. Other __________________ __________________________ | ❑ | ❑ | ❑ | ❑ |

[DISPLAY IF ANY ACTIVITIES MARKED IN Q17 ABOVE:]

**18. We are interested in how you have funded the HPV vaccination activities focused on advocacy and public policy that you indicated above. Below, please describe any federal or state agency, non-profit organization or other sources of funding for these activities. Please note which activities and year(s) (2013, 2014, or 2015) the funding is for.**

|  |
| --- |

[DISPLAY IF ANY ACTIVITIES MARKED IN Q13 ABOVE:]

**19. Did you have partners or collaborating organizations for the HPV vaccination activities focused on advocacy and public policy that you indicated above? These may include any federal or state agencies, non-profit organizations, local health departments, health systems or clinics, advocacy organizations, state or local cancer or immunization coalitions, pharmacies, pharmaceutical companies or other organizations.**

🔾 No, we did NOT have partners or collaborators; my organization did this work on our own

🔾 Yes, we did have partners or collaborators. Please specify the names of these partners or collaborators and the activities on which they worked with you:

|  |
| --- |

[DISPLAY IF ANY ACTIVITIES MARKED IN Q13 ABOVE:]

**20. When you implemented the activities focused on advocacy and public policy that you indicated above, how often did you or your organization encounter any of the following barriers?**

|  | **Never**  0% | **Rarely**  1-25% | **Some-times**  26-50% | **Often**  51%-75% | **Always**  >75% |
| --- | --- | --- | --- | --- | --- |
| a. Concerns about vaccine safety | O | O | O | O | O |
| b. Concerns about vaccine efficacy | O | O | O | O | O |
| c. Reluctance to discuss sexuality or sexually transmitted infections | O | O | O | O | O |
| d. Concern that vaccinated adolescents will practice riskier sexual behaviors | O | O | O | O | O |
| e. Lack of education/understanding about HPV infection | O | O | O | O | O |
| f. Lack of information about the HPV vaccine | O | O | O | O | O |
| g. Belief that adolescents are not at risk for HPV infection | O | O | O | O | O |
| h. Belief that younger adolescents are too young for the HPV vaccination | O | O | O | O | O |
| i. Lack of knowledge that vaccine is a series of three shots | O | O | O | O | O |
| j. Concerns about logistical or other barriers to returning for series of three shots | O | O | O | O | O |
| k. Lack of provider recommendations for HPV vaccination | O | O | O | O | O |
| l. Belief that HPV vaccine is recommended only for girls | O | O | O | O | O |
| m. Belief that girls or women should be the ones to take preventative steps against cervical cancer | O | O | O | O | O |
| n. Cost of HPV vaccine for family | O | O | O | O | O |
| o. Concerns about the cost of HPV vaccine for providers | O | O | O | O | O |
| p. Concerns about the cost of HPV vaccine for publicly-funded programs | O | O | O | O | O |
| q. Concerns about the failure of some insurance companies to cover the cost of vaccination | O | O | O | O | O |
| r. Barriers due to HPV vaccination not being required for school attendance | O | O | O | O | O |

**21. Did you encounter any other barriers besides those just mentioned?**

|  |
| --- |

**F. Now we would like to ask you some questions about you and your organization.**

**22. What is your position or role in your organization? Please mark all that apply.**

🔾 Public health professional

🔾 Health care provider

🔾 Educator

🔾 Advocate

🔾 Member/staff of a community-based organization

🔾 Researcher/academic staff

🔾 Student or intern

🔾 Insurer

🔾 Quality improvement

🔾 Policy maker

🔾 Journalist/author

🔾 Other _________________________

**23. What type of organization do you work for? (Please mark all that apply.)**

❑ Insurance plan

❑ Local public health department or program

❑ State public health department or program

❑ Local school district

❑ Other state or local government (not public health department or school district)

❑ Health care provider/health system

❑ Community-based organization

❑ College or university

❑ Pharmaceutical company

❑ Other _________________________

**24. Are you or your organization a member of your local or regional immunization coalition?**

🔾 No

🔾 Yes

🔾 I don’t know

**25. Does your organization currently access the Wisconsin Immunization Registry?**

🔾 No

🔾 Yes

🔾 I don’t know

**26. Does your organization currently use RECIN to track immunizations?**

🔾 No

🔾 Yes

🔾 I don’t know

**27. Does your organization currently use an EHR (Electronic Health Record) to track immunizations?**

🔾 No

🔾 Yes

🔾 I don’t know

**28. What is the interest level of your organization in conducting new activities (activities not already mentioned above as planned for 2015) to promote HPV vaccinations? These activities could focus on adolescents and their parents, clinical and health professionals, communities or health systems, advocacy or public policy, or other activities.**

🔾 High interest

🔾 Medium interest

🔾 Low interest

🔾 No interest

🔾 I don’t know

**29. What is the capacity (time and willingness) of your organization to take on new activities (activities not already mentioned above as planned for 2015) to promote HPV vaccinations? These activities could focus on adolescents and their parents, clinical and health professionals, communities or health systems, advocacy or public policy, or other activities.**

🔾 High capacity

🔾 Medium capacity

🔾 Low capacity

🔾 No capacity

🔾 I don’t know

**30. Does your organization have at least one staff member who either currently serves as a lead/champion for a project to increase HPV vaccination rates or who could do so in the future?**

🔾 No

🔾 Yes

🔾 I don’t know

[IF Q30 = YES, THEN DISPLAY Q30B.]

30b. Please provide the name and contact information for this lead/champion so that we may contact them about future activities to increase HPV vaccination rates in Wisconsin.

First Name ____________________

Last Name ____________________

Position ____________________

Organization ____________________

E-mail Address ____________________

Phone Number ____________________

**Additional Comments and Contacts**

**31. Is there anything else you would like to share about your organization’s work to promote HPV vaccination?**

|  |
| --- |

**32. Do you have any additional thoughts on barriers to increasing HPV vaccination rates in Wisconsin or ideas for activities that would be useful in increasing HPV vaccination rates?**

|  |
| --- |

**33. May we contact you or your organization if we have questions about your answers on this survey or would like more information about the activities you describe?**

🔾 Yes

🔾 No

**34. Would you like us to contact you or your organization to discuss possible future HPV vaccination activities?**

🔾 Yes

🔾 No

**35. Please provide the contact information for the best person to contact (you or a colleague at your organization) in the future about increasing HPV vaccination rates in Wisconsin.**

First Name ____________________

Last Name ____________________

Position ____________________

Organization ____________________

E-mail Address ____________________

Phone Number ____________________
